# Bidirectional relationship between sleep and sedentary behavior in adults with overweight or obesity: A secondary analysis

**DOI:** 10.1093/sleepadvances/zpab004

**Published:** 2021-03-26

**Authors:** Christopher C Imes, Zhadyra Bizhanova, Christopher E Kline, Bonny Rockette-Wagner, Eileen R Chasens, Susan M Sereika, Lora E Burke

**Affiliations:** 1 School of Nursing, University of Pittsburgh, Pittsburgh, PA; 2 Graduate School of Public Health, University of Pittsburgh, Pittsburgh, PA; 3 Department of Health and Human Development, School of Education, University of Pittsburgh, Pittsburgh, PA

**Keywords:** actigraphy, exercise, statistics

## Abstract

**Study Objectives:**

The relationships between daytime sedentary behavior and that night’s sleep and sleep and next day’s sedentary behavior are unknown. The purpose of this analysis was to examine these potentially bidirectional associations.

**Methods:**

This study was a secondary analysis of baseline data from an ecological momentary assessment study to determine the triggers for dietary lapses during a weight loss intervention. Sedentary behavior, physical activity, and sleep were objectively measured using accelerometers. Linear mixed modeling was used to examine the bidirectional multivariate associations between activity and sleep characteristics for each outcome examined separately. The models included sex, age, body mass index (BMI), education, and day of the week (weekday vs. weekend).

**Results:**

Participants were predominantly white (81.5%) and female (88.9%) with a mean age of 51.2 ± 10.6 years. Longer previous night’s total sleep time (TST) (*b* = −0.320, standard error [SE] = 0.060; *p* < .001) and being a weekend (*b* = −63.845, SE = 9.406; *p* < .001) were associated with less sedentary time the next day. More daytime sedentary time was associated with less wake after sleep onset (*b* = −0.018, SE = 0.008; *p* = .016), fewer awakenings (*b* = −0.010, SE = 0.004; *p* = .016), and less TST (*b* = −0.060, SE = 0.028; *p* = .029) that night.

**Conclusions:**

The bidirectional relationships between sedentary time and sleep characteristics are complex and may vary depending on participant characteristics and duration of sedentary and sleep time. Interventions to decrease sedentary behavior may benefit by targeting sleep duration and weekday activity.

**Statement of Significance**

The potentially bidirectional relationships between daytime sedentary behavior and that night’s sleep and sleep and next day’s sedentary behavior are unknown and need further exploration. In this analysis, previous night’s total seep time and type of day (i.e. weekday vs. weekend) predicted next day sedentary time. Additionally, daytime sedentary time predicted that night’s wake after sleep onset, awakenings, and total sleep time (TST). These findings have implications when developing interventions aimed at decreasing sedentary time and improving sleep.

## Introduction

Sedentary behavior, defined as any waking behavior characterized by energy expenditure ≤ 1.5 metabolic equivalents (METs) while in a sitting, reclining or lying posture [1, 2], is a highly prevalent behavior. Adults in the United States average over 5 hours of television watching per day in addition to one-and-a-half hours of computer and tablet time [3]. As a distinct behavior from physical activity, it is possible to meet the physical activity recommendations while still engaging in excessive sedentary behavior. Excessive sedentary time is an independent risk factor for all-cause mortality, diabetes mellitus, cardiovascular disease, and metabolic syndrome [4]; these risks remain after adjusting for physical activity [5].

Similarly, short sleep duration is prevalent. Commonly defined as sleeping less than 7 hours in a 24-hour period [6], over one-third of US adults report short sleep duration [7]. It is associated with numerous health outcomes, including all-cause mortality, diabetes mellitus, cardiovascular disease, stroke, metabolic syndrome, and obesity [6, 8, 9].

Excessive sedentary time and short sleep duration are associated with the same cardiometabolic disorders. However, unlike other risk factors for these conditions, such as age, sex, and family history, sedentary time and sleep duration are modifiable [10]. Physical activity and sleep influence each other through reciprocal physiological and psychological pathways, but much less is known about the relationship between sedentary behavior and sleep [11]. A better understanding of this potentially bidirectional relationship is needed to develop evidence-based interventions to reduce cardiometabolic risk factors and disorders.

Furthermore, day of the week (i.e. weekdays vs. weekends) may influence sedentary time and sleep. Weekends are often associated with increased physical activity [12, 13] and differences in sleep and wake patterns [14]. To develop and design interventions with the most impact, it is important to examine the differences in both sedentary behavior and sleep characteristics on weekday and weekends.

The purpose of this secondary analysis was to examine, in a sample of adults with overweight or obesity participating in a behavioral weight loss intervention, (1) the associations between the type of day (i.e. weekday vs. weekend) and activity (sedentary behavior and moderate-to-vigorous physical activity [MVPA]) and sleep characteristics and (2) the bidirectional associations between the previous night’s sleep and the next day’s activity and daytime activity and that night’s sleep. We hypothesized that (1) weekdays are associated with more sedentary time, less physical activity, and shorter sleep duration compared to weekends, (2) shorter sleep duration and greater wake after sleep onset (WASO) are associated with more sedentary behavior and less MVPA the next day, and (3) greater daytime sedentary behavior and less daytime MVPA are associated with shorter sleep duration and greater WASO that night.

## Methods

### Study design

This study was a secondary analysis of the objectively measured baseline activity and sleep data from the EMPOWER Study. The EMPOWER Study used ecological momentary assessment to determine the triggers for dietary lapses and relapses during a weight loss intervention. All participants received a behavioral weight loss intervention over 12 months. Details of the EMPOWER Study have been published previously [15].

Inclusion criteria for the EMPOWER Study included: (1) 18 years of age or older; (2) a body mass index (BMI) of 27 to 44 kg/m^2^; and (3) no participation in a weight loss program in the previous three months. Exclusion criteria included: (1) any medical condition that could confound the study findings (e.g. pregnancy, post-bariatric surgery, or diabetes); (2) planned pregnancy in the next year; (3) current treatment for serious mental illness; and (4) alcohol intake >3 drinks per day. A diagnosis of obstructive sleep apnea (OSA) and the use of sleep aids were collected at baseline but were not used to determine an individual’s eligibility for the study. The study was approved by the University of Pittsburgh’s Institutional Review Board and written informed consent was obtained before the initiation of research procedures. The only additional criteria for inclusion in this secondary analysis was at least two weekdays and two weekend days of overlapping accelerometer-based physical activity and sleep data.

### Measures

Participants completed a 25-item demographic questionnaire that included age, sex, race, marital status, and years of formal education. Race was dichotomized as “white” and “non-white.” Marital status was dichotomized as “married or living with a partner/significant other” and “never married, widowed, separated, or divorced.” Education was dichotomized as “12 years (high school level education) or less” and “>12 (some college, college degree, or master’s/graduate degree).”

Menopause status was obtained from a medical history form with responses including pre-menopausal, peri-menopausal, post-menopausal, and surgically induced menopause.

Weight was measured in pounds using a digital scale (Tanita Corp., Arlington Heights, IL) with participants wearing light clothes with their shoes removed. Height was obtained using a wall-mounted stadiometer. BMI (kg/m^2^) was calculated using weight and height.

Sedentary behavior and physical activity were assessed using a waist-worn accelerometer (ActiGraph GT3x, ActiGraph Corp., Pensacola, FL) for 7 days. Data from participants with at least 10 hours/day of wear time during waking hours was considered valid. Activity was recorded in one-second epochs and compressed to 60-second epochs using the ActiLife software (version 6.13.3, ActiGraph Corp., Pensacola, FL). Raw output in hertz was converted to monitor “counts” (per minute) with proprietary algorithms within the ActiLife software. Previously validated cut-points of 0–149 counts and ≥2690 counts were used to determine time spent in sedentary behavior and MVPA, respectively [16]. Additionally, daily steps were recorded by the accelerometer.

Sleep was objectively assessed using a watch-like device (Actiwatch 2, Philips Respironics, Murrysville, PA) worn on the wrist of the non-dominant hand for up to 14 days. A brief diary was completed concurrently each day and provided information on the participant’s bedtime and waketime. Rest intervals were manually established using standardized procedures that considered multiple inputs (i.e. event markers, diaries, activity, and light). Sleep/wake status for each 30-second epoch of data was computed using manufacturer-provided software (Actiware v.6.0, Philips Respironics, Murrysville, PA) utilizing settings of 5 immobile minutes for sleep onset, 0 immobile minutes for sleep offset, and a wake threshold of 40 counts [17, 18]. The following variables were considered for analysis: total sleep time (TST), WASO, the number of awakenings, sleep fragmentation, and sleep efficiency. TST was defined as the time, in minutes, scored as sleep within each main rest interval. WASO was defined as the total number of minutes in a sleep interval scored as wake by the software. The number of awakenings was the total number of discrete continuous periods scored as wake in a sleep interval. The sleep fragmentation index was calculated as the sum of percent mobile and percent immobile bouts less than one-minute in duration within a rest interval. Lastly, sleep efficiency was defined as the percentage of the rest interval that was scored as sleep.

The data will be shared on reasonable request to the senior author.

### Statistical analysis

Weekday physical activity included activity on Monday, Tuesday, Wednesday, Thursday, or Friday. Weekday sleep, the sleep that occurred the night before a weekday, included sleep on Sunday, Monday, Tuesday, Wednesday, or Thursday night. Weekend physical activity included activity on Saturday or Sunday. Weekend sleep, the sleep that occurred before a weekend day, included sleep on Friday or Saturday night.

Data were screened for missingness, outliers, and normality. Descriptive analyses were conducted using SPSS version 26 (IBM Corp., Armonk, NY). The significance level was set at 0.05 for two-sided hypothesis testing. Descriptive statistics for continuous variables were reported as mean ± standard deviation (SD). Categorical variables were described using frequency counts and percentages. The characteristics of the individuals included in the present analyses and those excluded due to not meeting the monitor wear requirements were compared using paired *t*-tests for continuous data and chi-squares or Wilcoxon rank-sum test for categorical data. To examine weekday and weekend differences in activity and sleep characteristics, paired *t*-tests were performed.

Linear mixed modeling (LMM) was used to examine the bidirectional univariate and multivariate associations between activity and sleep characteristics for each outcome examined separately (i.e. previous night’s sleep on next-day activity, and activity on that night’s sleep) using SAS version 9.4 (SAS, Cary, NC). Each univariate model included the number of measurements in the repeated and class statements and compound symmetry in the covariance structure. All multivariate models included sex, age, BMI, education, and day of the week (weekday vs. weekend). The potential confounders were examined using the results from univariate and multivariate models (beta coefficients, standard error [SE], *R*^2^, and *p*-values) at alpha level of 0.10. Additionally, including race and relationship status in the multivariate models did not change the estimates, *R*^2^, and *p*-values for any of the outcome variables; thus, race and relationship status were not included in the analysis.

To test multicollinear relationships between the previous night’s sleep characteristics and the next day’s activity, the correlations between TST, WASO, awakenings, sleep fragmentation, and sleep efficiency were examined. WASO, sleep efficiency, awakenings, and sleep fragmentation were highly correlated (*r* > 0.74, *p* < .001). As a result, only TST and WASO were included in these models as predictor variables. A similar approach was used to test multicollinear associations between that day’s activity and that night’s sleep. The correlations between sedentary time, MVPA, and mean daily steps were examined. MVPA and daily steps were also highly correlated (*r* > 0.68, *p* < .001). Thus, only sedentary time and MVPA were included as predictor variables in these models.

As noted in our previous work using these data, a significant percentage of the participants had undiagnosed OSA at baseline [19]. Separate analyses (not shown) were conducted on the subset of participants with OSA data. The inclusion of apnea-hypopnea index, a measure OSA severity, did not change the reported associations.

Lastly, separate analyses examined the potential influence of menopausal status on these associations. In these analyses, restricted to females only, sex (male or female) was replaced by menopause status (pre-menopausal, peri-menopausal, post-menopausal, or surgically induced menopause).

## Results

### Participant characteristics

Of the 151 individuals in the EMPOWER study, 108 had at least 2 weekdays and 2 weekend days with overlapping activity and sleep data. The mean number of overlapping weekday data per participant was 3.7 days. The sample was predominantly white (81.5%), female (88.9%), pre-menopausal (41.7%) or post-menopausal (32.3%), 50.61 ± 10.64 years of age, and had a BMI of 33.94 ± 4.63. The majority had received some college or higher level of education (88.0%) and were married or living with a significant other (63.9%). Those excluded from these analyses (*n* = 43) due to insufficient activity and sleep data did not differ from those included in the analyses on any of the sociodemographic characteristics (Table 1).

**Table 1.** Demographics characteristic of individuals included in the secondary analysis compared to those excluded due to insufficient activity and sleep data

	Included (*n* = 108)	Excluded (*n* = 43)	
Characteristic	Mean ± SD or % (*n*)	Mean ± SD or % (*n*)	*p*
Age, years	51.2 ± 10.6	52.7 ± 9.0	.258
Sex, female	88.9% (*n* = 96)	95.7% (*n* = 45)	.218
Menopause status			
Pre-menopausal	41.7% (*n* = 40)	20.0% (*n* = 9)	.101
Peri-menopausal	18.8% (*n* = 18)	26.7% (*n* = 12)	
Post-menopausal	32.3% (*n* = 31)	40.0% (*n* =18)	
Surgically induced menopause	6.3% (*n* = 6)	4.4% (*n* = 2)	
Race, white	81.5% (*n* = 88)	76.6% (*n*= 36)	.334
BMI, kg/m^2^	33.9 ± 4.6	34.3 ± 4.5	.628
Relationship status, married or living with significant other	63.9% (*n* = 69)	58.7% (*n* = 27)	.621
Education, some college or higher	88.0% (*n* = 95)	91.5% (*n* = 43)	.632

SD, standard deviation; BMI, body mass index.

### Weekday and weekend differences

The activity and sleep characteristics of the participants differed based on weekday and weekend status (Table 2). On weekdays, participants engaged in more minutes of MVPA while also having greater sedentary time than on weekends. Regarding sleep characteristics, TST was shorter on weekday nights compared to weekend nights. Minutes of WASO and the number of awakenings per night were fewer on weekday nights (Table 2). However, there was no statistically significant difference in sleep fragmentation or sleep efficiency between weekday and weekend nights.

**Table 2.** Weekday and weekend differences in physical activity and sleep characteristics

Characteristic	All valid days	Weekdays	Weekends	p*
Steps per day	6248 ± 2998	6393 ± 2518	5970 ± 3002	.081
MVPA, minutes	11.8 ± 17.0	12.9 ± 16.7	9.7 ± 17.5	.026
Sedentary time, minutes	638.8 ± 131.2	669.3 ± 124.0	580.0 ± 127.7	<.001
Sleep efficiency, %	89.1% ± 6.2%	89.3% ± 5.7%	88.7% ± 7.1%	.234
WASO, minutes	43.7 ± 26.5	40.7 ± 22.6	49.4 ± 31.9	<.001
Awakenings per night	33.7 ± 26.5	31.9 ± 14.3	37.0 ± 14.6	<.001
TST, minutes	409.8 ± 83.7	392.0 ± 75.5	443.9 ± 88.0	<.001
Sleep fragmentation	18.7 ± 10.1	18.1 ± 9.2	19.7 ± 11.5	.063

MVPA, moderate-to-vigorous physical activity; WASO, wake after sleep onset; TST, total sleep time. Data shown are mean ± standard deviation.

*Paired *t*-test differences between weekday and weekend days.

### Overall associations between previous night’s sleep and next-day activity

In the model that examined the association between sleep characteristics and next-day sedentary time, greater TST (*b* = −0.32, SE = 0.06; *p* < .001) and being a weekend (*b* = −63.85, SE = 9.41; *p* < .001) were each jointly associated with less sedentary time after adjusting for WASO, sex, age, BMI, and education (Table 3). Thus, 60 minutes of additional TST was associated with 19.2 minutes less sedentary time the next day. In separate models for next day’s steps and MVPA, neither WASO nor TST were associated with the next day’s total step count or MVPA. Being a female (*b* = −16.54, SE = 3.20; *p* < .001), having a higher BMI (*b* = −0.44, SE = 0.22; *p* = .04), and weekends (*b* = −3.44, SE = 1.32; *p* = .01) were associated with less MVPA (Table 3).

**Table 3.** Multivariate relationships of the previous night’s sleep with the next day’s physical activity among males and females (*N* = 108)

Next-day physical activity outcome						
	Mean daily steps		MVPA time		Sedentary time	
Predictor	*b* (SE)	*p*	*b* (SE)	*p*	*b* (SE)	*p*
WASO	−7.07 (4.21)	.094	−0.04 (0.03)	.105	−0.34 (0.19)	.082
TST	−1.52 (1.30)	.245	0.02 (0.01)	.064	−0.32 (0.06)	<.001
Sex, female	−681.68 (600.32)	.259	−16.54 (3.20)	<.001	−13.71 (27.31)	.617
Age, years	−12.73 (18.03)	.481	−0.18 (0.10)	.066	1.12 (0.82)	.175
BMI, kg/m^2^	−108.40 (40.42)	.008	−0.44 (0.22)	.044	1.03 (1.82)	.577
Education, some college or higher	272.21 (561.30)	.629	4.11 (2.98)	.171	−23.48 (25.53)	.360
Weekend, Yes	−339.34 (203.67)	.099	−3.42 (1.32)	.011	−63.85 (9.41)	<.001

MVPA, moderate-to-vigorous physical activity; SE, standard error; WASO, wake after sleep onset; TST, total sleep time; BMI, body mass index

### Overall associations between daytime activity and that night’s sleep

In the model that examined daytime activity on that night’s WASO, more sedentary time (*b* = −0.02, SE < 0.01; *p* = .02) was associated with less WASO after adjusting for MVPA, sex, age, BMI, education, and weekend status (Table 4). Similarly, in the separate model that examined how daytime activity predicted awakenings, more sedentary time (*b* = −0.01, SE < 0.01; *p* = .02) was associated with fewer awakenings while being a weekend day (*b* = 1.92, SE = 0.96; *p* = .05) was associated with more awakenings after adjusting for MVPA, sex, age, BMI, education, and weekend status (Table 4). For the model that examined the associations between daytime activity and TST, more sedentary time (*b* = −0.06, SE = 0.03; *p* = .03) was associated with less TST while being female (*b* = 43.83, SE = 15.66; *p* < .01) was associated with greater TST after adjusting for MVPA, age, BMI, education, and weekend status (Table 4). In separate models, neither MVPA nor sedentary time were associated with that night’s sleep efficiency or sleep fragmentation index. Thus, 60 minutes of additional sedentary time was associated with 1.2 minutes less WASO and 3.6 minutes greater TST.

**Table 4.** Multivariate relationships of daytime activity with that night’s sleep at baseline among males and females (*N* = 108)

That night’s sleep outcome										
	Sleep efficiency		WASO		Awakenings		TST		Sleep fragmentation	
Predictor	*b* (SE)	*p*	*b* (SE)	*p*	*b* (SE)	*p*	*b* (SE)	*p*	*b* (SE)	*p*
MVPA	−0.009 (0.013)	.465	−0.010 (0.057)	.863	−0.055 (0.031)	.075	−0.197 (0.209)	.346	−0.006 (0.021)	.776
Sedentary time	0.003 (0.002)	.069	−0.018 (0.008)	.016	−0.010 (0.004)	.016	−0.060 (0.028)	.029	−0.004 (0.003)	.180
Sex, female	−0.063 (1.472)	.966	5.062 (5.735)	.379	2.812 (3.497)	.423	43.834 (15.655)	.006	−0.691 (2.361)	.771
Age, years	0.021 (0.044)	.636	−0.161 (0.172)	.348	−0.141 (0.105)	.180	−0.267 (0.464)	.565	0.027 (0.071)	.770
BMI, kg/m^2^	−0.050 (0.098)	.610	0.212 (0.383)	.580	0.300 (0.234)	.200	1.625 (1.039)	.119	0.243 (0.158)	.124
Education, some college or higher	0.813 (1.371)	.555	−0.409 (5.330)	.939	0.405 (3.258)	.901	16.552 (14.414)	.253	−0.574 (2.199)	.795
Weekend, Yes	−0.830 (0.411)	.046	2.754 (1.801)	.129	1.924 (0.963)	.048	7.493 (6.925)	.282	1.101 (0.662)	.099

WASO, wake after sleep onset; TST, total sleep time; BMI, body mass index; SE, standard error; MVPA, moderate-to-vigorous physical activity.

### Associations between previous night’s sleep and next-day activity – Females only

When the analyses were conducted using the data from the female participants only with menopause status included, the results changed slightly. Greater TST (*b* = 0.02, SE = 0.01; *p* = .027) and having a lower BMI (*b* = −0.42, SE = 0. 21; *p* = .04) were associated with greater time spent in MVPA (Supplementary Table 1). While weekends were still associated with less MVPA and sedentary time, these associations become statistically non-significant.

### Associations between daytime activity and that night’s sleep—females only

When the analyses were conducted using the data from the female participants only with menopause status included, sedentary time was no longer associated with WASO and awakenings (Supplementary Table 2).

## Discussion

Our results clearly demonstrate an association between weekday and weekend activity and sleep. In the study’s sample of mostly middle-aged women who had overweight or obesity, MVPA was greater on weekdays than on weekends. This partially goes against the study’s first hypothesis that weekdays are associated with more sedentary time and less physical activity. Work-related activities may be contributing to this finding [12] and may vary according to the job-required physical activity demand. In our sample, sedentary time was less on weekends than on weekdays. This suggests that there is an increase in light-intensity physical activity during weekends, possibly associated with a preponderance of sedentary jobs during the weekdays. Pettee Gabriel and colleagues [13] also reported a decrease in sedentary time on weekends. Unfortunately, information on job type was not collected in the parent study. Supporting the study’s first hypothesis, we found that TST was approximately 50 minutes longer on weekend nights compared to weekday nights.

When interpreting the results, it is important to remember that every day is a fixed length of 24 hours. Thus, an increase in one behavior necessitates a decrease in another behavior [20]. In our sample, as both sedentary time and time spent engaged in MVPA increased on weekdays, the amount of available time for sleep decreased. On weekends, sleep duration increased and the amount of available time for sedentary behavior and MVPA decreased.

The results of our study show the complex relationships between activity patterns and sleep. The previous night’s TST was negatively associated with sedentary time; for each additional minute of TST, the next day’s sedentary time decreased by 0.32 minutes. However, we did not observe associations between TST or WASO with steps or MVPA in the analyses that included both males and females. However, in the analyses that included the females only, TST was associated with MVPA. These results partially support the study’s second hypothesis.

Our findings are similar to those by Pettee Gabriel and colleagues [21], who found that time in bed, which was used to estimate sleep duration, was negatively associated with next-day sedentary time. Their study relied on self-reported sleep from a sample of 10 086 females from the Women’s Health Study with a mean age of 71.6 (± 5.7) years. Similarly, a cross-sectional study of over 6000 European adults found that short sleep duration, defined in the study as <6 hours/night, was associated with greater screen time, a contributor to overall sedentary time, compared to normal sleepers [22]. In contrast, a study by Mitchell and colleagues [23] does not support our findings. Their study used objective measures of physical activity and sleep and had a similar sample in terms of age, sex, and race. No association was found between TST and the next day’s sedentary time. The sample in their study engaged in approximately 534 ± 90 minutes of sedentary time/day compared to 639 ± 131 minutes/day in our sample.

The results for the associations between daytime activity and that night’s sleep were mixed. Sedentary time was negatively associated with WASO, that night’s awakenings, and TST. It is important to note that, while these findings are statistically significant, their clinical significance is questionable (e.g. 60 greater minutes of sedentary time was associated with 1.2 minutes less WASO and 3.6 minutes greater TST). We found no associations between daytime MVPA and that night’s sleep. Again, these results partially support the study’s third hypothesis.

Our findings regarding daytime activity and that night’s WASO are contradictory to the results reported by Dzierzewski and colleagues [24]. While they did not examine sedentary time, they found that less physical activity was associated with greater WASO [24]. Differences in the studies’ participants may be contributing to the contradictory findings. The participants in our study were younger, had a higher BMI, and were less active than the participants in Dzierzewski’s study [24].

Numerous previous studies support an association between daytime activity and that night’s sleep. Pettee Gabriel and colleagues reported that less sedentary time and higher accelerometer counts during the day were associated with a reduced likelihood of reporting short sleep duration that night [21]. Similarly, Best and colleagues [25] found that greater daytime physical activity was associated with longer sleep duration that night.

Among middle-aged and older adults, Mesas and colleagues [26] found associations between physical activity and multiple sleep characteristics. Specifically, they found that 500 to 1500 MET-minutes/week was associated with decreased risk for TST < 6 hours, WASO > 60 minutes, and sleep efficiency < 80%. However, when MET-minutes/week was > 1500, the risks for TST <6 hours and sleep efficiency < 80% were not statistically significantly different from those engaging in < 500 MET-minutes/week of activity [26]. A meta-analysis by Kredlow and colleague [27] revealed that both acute and regular exercise were associated with improved TST. In our sample, we did not find associations between daytime MVPA and that night’s sleep. This finding could be due to the low levels of MVPA. It is possible that there is a minimum duration of MVPA that is needed to affect sleep and that was not met by the participants in our study. Additionally, our analyses examined the daily bidirectional associations between sleep and daytime activity. Therefore, the effect of regular activity or habitual physical activity on sleep and vice versa were not examined.

Looking specifically at sedentary behavior, Buman and colleagues examined the associations between sitting, in general, and television viewing while sitting and sleep in a sample of 843 adults [28]. They found that both sitting and television viewing while sitting were associated with poor sleep quality and that television viewing while sitting was associated with longer sleep onset latency and waking up earlier than desired in the morning [27]. While we found an association between sedentary time and TST, sleep quality and the activities that occurred during sedentary time were not collected as part of our study.

There were differences in the analyses that included all the participants and the analyses that included only the female participants and menopause status. In the analyses restricted to females only, we observed an association between the previous night’s TST and next day’s MVPA. The associations between that day’s sedentary time and that night’s WASO and awakenings were only found in the analyses that included all the participants. Peri- and post-menopausal status are associated with a higher prevalence of sleep disorders, sleep fragmentation, and hot flashes resulting in a greater number of awakenings [29]. These changes may account for the different findings in the analyses conducted in the full versus female-specific sample.

The findings from the study, as a whole, support the idea that sleep is multifaceted and complex. Looking at a single sleep characteristic does not adequately capture the whole sleep experience. The use of a composite sleep score, such as the RuSATED sleep health framework which incorporates sleep regularity, satisfaction, alertness, timing, efficiency, and duration, should be considered for future studies examining the bidirectional relationships between physical activity, sedentary behavior, and sleep [30]. For example, a recent study by Kubala and colleagues found a positive association between minutes of MVPA and better sleep health assessed using the composite RuSATED score [31].

While some of the findings from our analyses are seemingly inconsistent with the results of previous work, the participants in the different studies varied considerably. Thus, our results may be used to inform the development of interventions for middle-aged women with overweight or obesity. Increased TST, especially on weekday nights, may decrease next-day sedentary time. Similarly, decreased sedentary time may have a small effect on that night’s TST. Interventions aiming to improve sleep may need to focus on weekday habits, whereas interventions aiming to improve physical activity may need to focus on weekend habits.

Limitations of the study include a relatively homogenous sample, the variability in the amount of weekday data, and the study being a secondary analysis. The study was limited to adults with overweight or obesity who were seeking a weight loss intervention. Given the mostly white, female, middled-aged, well-educated sample, these results are relevant to this specific population and not generalizable. This was a secondary analyses and important factors affecting sleep (e.g. the use of sleep aids) were not collected concurrently with the sleep data. Data on napping were not included in the analyses. Dietary self-monitoring was included as part of the weight loss intervention, but alcohol intake may have been unreported. Thus, our analyses did not include alcohol intake as a variable. Sleep quality, an important factor that assesses the perceived quality of one’s sleep, was not collected on a nightly basis. However, a major strength of the study is the use of objectively measured sleep and activity data collected concurrently.

## Conclusion

These results demonstrate the associations between weekday and weekend activity and sleep characteristics in our sample. This knowledge can be used to design and enhance interventions aimed at decreasing sedentary time, increasing physical activity, and improving sleep. Furthermore, results from the study suggest complex, bidirectional relationships between sedentary time, physical activity, and sleep characteristics. While these findings were statistically significant, their clinical relevance is unknown. Additionally, the nature of these relationships may vary depending on numerous factors such as age, body composition, menopausal status and duration of sedentary time, MVPA, TST, and WASO. Additional research is needed to further expand our knowledge of these relationships and factors.

## Supplementary Material

zpab004_suppl_Supplementary_Tables
