# Supplementary material for: Bidirectional relationship between sleep and sedentary behavior in adults with overweight or obesity: A secondary analysis
Source: Sleep Adv. 2021 Mar 26;2(1):zpab004. doi: 10.1093/sleepadvances/zpab004 (PMC8038645; doi:10.1093/sleepadvances/zpab004)
Supplement: zpab004_suppl_Supplementary_Tables [file zpab004_suppl_supplementary_tables.docx]

**Supplementary Table 1.** Multivariate relationships of the previous night’s sleep with the next day’s physical activity among females only with reported menopausal status (n = 95)

| Next-day physical activity outcome | | | | | | |
| --- | --- | --- | --- | --- | --- | --- |
| Predictor | Mean Daily Steps | | MVPA Time | | Sedentary Time | |
|  | b (SE) | p | b (SE) | p | b (SE) | p |
| WASO | -5.59  (4.29) | 0.193 | -0.03 (0.03) | 0.171 | -0.19 (0.20) | 0.346 |
| TST | -1.38  (1.34) | 0.306 | 0.02  (0.01) | 0.027 | -0.34 (0.06) | <.001 |
| Menopausal status    Pre-    Peri-    Post-    Surgically induced  menopause | Ref.  -1153.45  (624.91)  -1238.03  (696.34)  819.30  (954.30) | 0.031  0.068  0.079  0.393 | Ref.  -2.76  (3.08)  -2.78  (3.43)  2.96  (4.69) | .446  .372  .420  .530 | Ref.  22.13  (29.25)  38.99  (29.25)  48.19  (44.67) | .638  .451  .235  .284 |
| Age, years | 24.08 (30.73) | 0.434 | -0.09 (0.15) | 0.551 | -0.31  (1.44) | 0.828 |
| BMI, kg/m^2^ | -104.80 (41.90) | 0.013 | -0.42 (0.21) | 0.041 | 1.26  (1.96) | 0.522 |
| Education, some college or higher | 570.35 (565.67) | 0.316 | 4.97 (2.78) | 0.078 | -19.56 (26.48) | 0.462 |
| Weekend, Yes | -747.79 (552.85) | 0.179 | -32.69 (3.33) | 0.422 | -35.53 (25.84) | 0.172 |

Abbreviations: MVPA: moderate-to-vigorous physical activity; SE: standard error; WASO: wake after sleep on-set; TST: total sleep time; BMI: body mass index

**Supplementary Table 2.** Multivariate relationships of daytime activity with that night’s sleep at baseline among females only with reported menopausal status (n = 95)

| That night’s sleep outcome | | | | | | | | | | | | | | |  |
| --- | --- | --- | --- | --- | --- | --- | --- | --- | --- | --- | --- | --- | --- | --- | --- |
| Predictor | Sleep efficiency | | WASO | | | Awakenings | | | TST | | | Sleep Fragmentation | | | |
|  | b (SE) | p | b (SE) | p | b (SE) | | p | b (SE) | | p | b (SE) | | p |  |  |
| MVPA | 0.0002 (0.02) | 0.989 | -0.02 (0.07) | 0.754 | -0.06 (0.04) | | 0.130 | -0.12 (0.24) | | 0.620 | -0.02 (0.02) | | 0.497 |  |  |
| Sedentary time | 0.003 (0.002) | 0.197 | -0.01 (0.01) | 0.112 | -0.01 (0.01) | | 0.130 | -0.06 (0.03) | | 0.048 | -0.004 (0.003) | | 0.221 |  |  |
| Menopausal status  Pre-  Peri-  Post-  Surgically  induced  menopause | Ref.  1.25 (1.55)  3.48 (1.72)  1.07 (2.37) | 0.189  0.422  0.046  0.653 | Ref.  -6.78 (6.11)  -12.34 (6.79)  -3.47 (9.35) | 0.287  0.270  0.073  0.711 | Ref.  -2.94 (3.88)  -7.89 (4.31)  -3.36 (5.94) | | 0.296  0.450  0.070  0.572 | Ref.  -4.00 (16.59)  -2.59 (18.43)  -25.36 (25.34) | | 0.746  0.810  0.889  0.320 | Ref.  -3.34 (2.49)  -5.55 (2.76)  -0.33 (3.81) | | 0.147  0.183  0.048  0.932 |  |  |
| Age, years | -0.10 (0.08) | 0.196 | 0.22 (0.30) | 0.457 | 0.06 (0.90) | | 0.747 | -0.30 (0.81) | | 0.711 | 0.21 (0.12) | | 0.086 |  |  |
| BMI, kg/m^2^ | -0.02 (0.10) | 0.870 | 0.09 (0.41) | 0.831 | 0.30 (0.26) | | 0.250 | 1.74 (1.12) | | 0.120 | 0.20 (0.17) | | 0.239 |  |  |
| Education, some college or higher | 0.81 (1.40) | 0.567 | 0.31 (5.5) | 0.956 | 0.62 (3.52) | | 0.861 | 15.33 (15.05) | | 0.311 | -0.17 (2.26) | | 0.940 |  |  |
| Weekend, Yes | -0.16 (1.18) | 0.893 | -0.49 (5.20) | 0.925 | -3.29 (2.76) | | 0.237 | -26.76 (18.06) | | 0.142 | -1.101 (1.87) | | 0.588 |  |  |

Abbreviations: WASO: wake after sleep on-set; TST: total sleep time; BMI: body mass index; SE: standard error; MVPA: moderate-to-vigorous physical activity
